# Lactoferrin and SIgA Concentrations in Human Milk of SARS-CoV–Infected Mothers—Polish Cohort Study

**DOI:** 10.3390/nu17111840

**Published:** 2025-05-28

**Authors:** Aleksandra Mołas, Jolanta Lis-Kuberka, Agnieszka Bzikowska-Jura, Aleksandra Wesołowska, Tengchuan Jin, Maciej W. Socha, Magdalena Orczyk-Pawiłowicz

**Affiliations:** 1 Laboratory of Human Milk and Lactation Research in Warsaw Medical University at Regional Human Milk Bank in the Specialistic Holy Family Hospital in Warsaw, Department of Medical Biology, Faculty of Health Science, Medical University of Warsaw, Litewska Str. 14/16, 00-575 Warsaw, Poland; aleksandra.molas@wum.edu.pl (A.M.); abzikowska@wum.edu.pl (A.B.-J.); 2 Division of Chemistry and Immunochemistry, Department of Biochemistry and Immunochemistry, Wroclaw Medical University, M. Skłodowskiej-Curie Str. 48/50, 50-369 Wroclaw, Poland; jolanta.lis-kuberka@umw.edu.pl (J.L.-K.); magdalena.orczyk-pawilowicz@umw.edu.pl (M.O.-P.); 3 National Key Laboratory of Immune Response and Immunotherapy, Division of Life Sciences and Medicine, University of Science and Technology of China, Hefei 230027, China; jint@ustc.edu.cn; 4 Department of Perinatology, Gynecology and Gynecologic Oncology, Faculty of Health Sciences, Collegium Medicum in Bydgoszcz, Nicolaus Copernicus University, Łukasiewicza 1, 85-821 Bydgoszcz, Poland; maciej.socha@cm.umk.pl; 5 Department of Obstetrics and Gynecology, St. Adalbert’s Hospital in Gdańsk, Copernicus Healthcare Entity, Jana Pawła II 50, 80-462 Gdańsk, Poland

**Keywords:** human milk, infant feeding in emergencies, breastfeeding in emergencies, lactation, SARS-CoV-2, COVID-19 pandemic, lactoferrin, SIgA, immunology of human milk

## Abstract

**Background:** Human milk (HM) provides critical immunological support to neonates, serving as a key component of passive immunity during early life. **Objectives:** The main aim of this cohort study was to compare the concentrations of lactoferrin (Lf), secretory immunoglobulin A (SIgA), C-reactive protein (CRP), and their ratios to total protein levels in the colostrum of postpartum women infected with SARS-CoV-2 and healthy controls. **Methods:** Colostrum samples (3–5 mL) were collected from 40 mothers (20 infected, 20 healthy) during the first wave of the COVID-19 pandemic in Poland. Concentrations of Lf, SIgA, and CRP were analyzed using ELISA, and total protein content was measured using the bicinchoninic acid assay (BCA). **Results:** The presence of specific anti-SARS-CoV-2 SIgA antibodies was assessed via cassette serological lateral flow detection tests. Significant differences were observed in Lf (*p =* 0.04) and SIgA (*p =* 0.03) concentrations, both lower in the COVID-19 group. Lactoferrin medians were 12.30 g/L (infected) and 14.95 g/L (healthy), and for SIgA: 9.15 g/L vs. 15.01 g/L, respectively. No significant difference was found in CRP levels. Interestingly, the Lf/Protein ratio was significantly higher in the infected group (*p* = 0.03), whereas the SIgA/Protein ratio did not differ. Furthermore, 75% of infected mothers had positive anti-SARS-CoV-2 SIgA results. These mothers also showed a higher Lf/Protein ratio. Among healthy controls, 90% had negative test results. **Conclusions:** These findings suggest a potential compensatory role of lactoferrin in the nonspecific immune response to SARS-CoV-2, though stress-related reductions in SIgA levels cannot be excluded.

## 1. Introduction

The protective and beneficial power of human milk (HM) against viral infections due to the richness of its constituents related to the immune system has been known for more than two decades [1,2]. HM is considered the gold standard of infant nutrition, offering a complex array of bioactive components that provide immunological protection during the critical early stages of an infant’s life [3]. Exclusive breastfeeding provides numerous benefits for both mothers and infants. For infants, it offers optimal nutrition, being specifically tailored to meet their developmental needs. HM enhances immune protection through antibodies and beneficial bioactive compounds, reducing the risk of infections, such as gastrointestinal and respiratory illnesses. It promotes healthy growth and development by providing essential nutrients, including proteins, fats, vitamins, and minerals, in the right proportions. Furthermore, exclusive breastfeeding supports the establishment of a healthy gut microbiome, which is crucial for overall health and immunity. It also helps reduce the risk of chronic conditions later in life, such as obesity, diabetes, and allergies. Additionally, the act of breastfeeding fosters a strong emotional bond between mother and infant, contributing to better mental and emotional health outcomes for both. For mothers, exclusive breastfeeding can aid in postpartum recovery, and reduce the risk of certain cancers. These benefits underscore the importance of promoting exclusive breastfeeding practices, especially during critical early stages of life. Recent epidemiological data indicate that the exclusive breastfeeding rate in Poland for infants up to 6 months of age is 41% [4] highlighting a significant area for improvement in infant health practices.

Among the many biologically active components of HM, SIgA, and lactoferrin (Lf) are essential mediators of antimicrobial and immunomodulatory activity [5,6,7]. Emerging evidence highlights their synergistic roles in preventing infections, promoting beneficial gut microbial growth, and supporting mucosal immunity in neonates and infants. This is crucial because of their immature immune system, particularly in mucosal tissues such as newborns’ gastrointestinal and respiratory tracts [1,2]. This issue is even more important during emergencies when the risk of infant diseases (mainly malnutrition) and death is significantly higher [8]. In recent years, Poland has encountered multiple crises. In the second half of 2024, floods affected various regions of the country, and the war in Ukraine that began in early 2022 generated a substantial refugee crisis. Nevertheless, the most significant impact on perinatal care stemmed from the COVID-19 pandemic, which began in 2020.

From March to August 2020, Polish recommendations concerning implementing the standard of perinatal care in case of maternal SARS-CoV-2 infection were changed three times. Unfortunately, considering this emergency, during the initial period of the pandemic (March–April 2020), practices supporting breastfeeding (e.g., skin-to-skin contact—SSC, rooming-in) were banned. In the case of mothers suspected or confirmed for COVID-19, even breastfeeding was strongly contradicted, mainly due to the concerns about transmission of the virus from an infected mother to her baby. In September 2020, the Polish recommendations changed, and mothers were allowed to breastfeed (if special precautions were taken, e.g., hand hygiene and use of face masks during breastfeeding) [9]. Current knowledge about the immunology of HM during the SARS-CoV-2 infection is higher, and we know that the risk of virus transmission during breastfeeding is extremely low. Centeno-Tablate et al. [10] in their living systemic review concluded that there is no evidence of virus transmission through HM. However, only a few studies have aimed to assess the immunological properties of HM, considering firstly the aspect of infection and secondly the stress of COVID-19 disease.

Secretory IgA is one of the most important and abundant immunoglobulins of HM, as it constitutes approximately 90% of all antibodies. The level of SIgA in HM is lactation-stage related, the highest values are observed in colostrum. SIgA has both antibacterial and antiviral properties, directly preventing microorganisms from binding to host cells [11]. Moreover, during the mother’s infection, immunoglobulins against specific pathogens appear in HM. It was proved that the HM of mothers after COVID-19 may contain anti-SARS-CoV-2-specific IgA, even when the infection was asymptomatic [12,13]. In turn, Lf has antimicrobial and immunomodulatory properties. Thanks to its high affinity for iron, Lf has antibacterial properties because it binds to the lipoproteins of bacterial cells and forms receptor complexes, thereby inhibiting their absorption of iron, crucial for proper growth [14]. Moreover, Lf has antiviral activity, especially at the early stage of infection [15,16]. Additionally, lactoferrin can reduce the risk of respiratory infections and its beneficial effect in the prevention and treatment of COVID-19 is confirmed [17].

Women in the perinatal and postpartum periods are exposed to a high level of stress. Hormonal fluctuations during this period, socioeconomic situation, and their environment may influence their psychological state. High levels of stress experienced by the mother not only make breastfeeding difficult but can affect the composition of maternal milk [18,19,20]. It has been observed that stress increases may affect the metabolism of hormones like cortisol, prolactin or melatonin. Studies point out that stress the amount of cortisol and negatively affects total fats and selected fatty acids in milk. Moreover, alternations in the nutritional value of stressed mothers’ milk, including amino acid composition and protein-bound amino acids were also reported [19,21]. Furthermore, studies have shown that the specific stress of mothers during pregnancy and childbirth negatively influences the immunological properties of breast milk. Women who had increased levels of cortisol in their saliva had lower amounts of SIgA in their milk [22].

Considering that HM contains bioactive factors that can prevent newborn infections, we aimed to compare the concentrations of Lf and SIgA in the colostrum of SARS-CoV-2-infected and healthy mothers at the time of delivery.

## 2. Materials and Methods

### 2.1. Study Design

This observational cohort study targeted women with active SARS-CoV-2 infection during their birth admission who were delivered at Polish hospitals. Considering the total number of births from April 2020 to October 2020 (200,660), the number of hospitals involved in the collection of biological material (8 from 333), and the level of positive results of COVID-19 in the perinatal period (12%), the minimal sample size was estimated as 75 participants, with a confidence interval of 5% (absolute ± %) and a level of confidence of 95% (alpha = 0.05). Of the 48 recruited mothers, we excluded women who had chromosomal abnormalities, extremely and very preterm birth (<32 weeks), uncontrolled diabetes, alcohol consumption, and cigarette smoke during pregnancy. Finally, 40 breastfeeding mothers participated in the study, and the sample size bias was 9.3%.

### 2.2. Characteristics of the Study Participants

All women gave birth during the initial period of the SARS-CoV-2 pandemic (from April to October 2020). Quantitative reverse transcription polymerase chain reaction (RT-qPCR)-confirmed coronavirus infection tests were performed in all women and when the result was positive, the mother was assigned to the study group (*n* = 20), in the case of a negative result, women were involved in the control group (*n* = 20). SARS-CoV-2 positive mothers experienced the disease mildly or asymptomatically and were recruited from eight different Polish hospitals (University Clinical Centre, Gdańsk, MSWiA in Warsaw, Independent Public Specialist Healthcare Centre, Puławy, Megrez SP. Z. o. o. Provincial Specialist Hospital in Tychy, Independent Public Specialist Healthcare Centre, Kędzierzyn-Koźle, Gynecological Obstetric Clinical Hospital of Poznan University of Medical Sciences, Regional Hospital in Racibórz, Medical Centre in Łańcut), whereas healthy mothers gave birth in St. Adalbert’s Hospital in Gdańsk, Copernicus Healthcare Entity. Women in the symptomatic group experienced respiratory symptoms such as runny nose and rhinitis.

### 2.3. Milk Collection and Sample Pre-Treatment for Analysis

Milk samples were collected by a qualified nurse after obtaining written consent from the mother, following a protocol for the collection of milk from a mother ill or suspected of being infected with SARS-CoV-2 in a hospital setting [23]. All samples were collected during the woman’s stay in the maternal ward, at the first week after delivery, between 3 and 7 days of lactation. The maximum sample volume taken within several sessions per day was 10 mL, and the usual volume was between 3 and 5 mL, and was only provided for the study in the case of securing their own baby’s nutritional. The expressed milk was first stored at 4 °C for up to 12 h and then frozen and stored at −80 °C for further analysis. After defrosting, the collected HM samples were centrifuged at 3500× *g* at 4 °C for 35 min to remove fat and cells that may interfere with the analysis. The obtained defatted milk samples (skim milk) were stored at −20 °C [24].

### 2.4. Protein Concentration

The protein concentration in skim colostrum samples was determined with the Bicinchoninic Acid Protein Assay Kit (Sigma, St. Louis, MO, USA), according to a procedure reported previously [25]. For protein determination 25 μL of diluted colostrum samples and bovine albumin as a standard [from 0.1 to 1.0 mg/mL]. Afterward, 200 μL of bicinchoninic acid working solution was added, and the plate was incubated at 37 °C for 35 min. In the next step, the obtained absorbance was measured in a Synergy LX Multi-Mode Reader (BioTek Instruments, Inc., Winooski, VT, USA) at 560 nm. All defatted samples were assayed at two different colostrum sample dilutions (25- and 50-fold), each in duplicate. The intra-assay and inter-assay coefficients of variation were 0.5% and 1.5%, respectively.

### 2.5. Skim Milk Lactoferrin and SIgA Concentration

The concentrations of analyzed parameters, namely SIgA and Lf in skim milk samples, were quantified by the enzyme-linked immunosorbent assay (ELISA) according to the procedure presented previously [24,26]. Briefly, for all determinations, microtiter plates (Nunc International, Naperville, IL, USA) were used. Standard curves were prepared using commercially available standards of human colostrum IgA (detection range: from 1.56 to 50 ng/100 μL) (Sigma, St. Louis, MO, USA) and for Lf isolated from HM (detection range: from 1.56 to 50 ng/100 μL) (Sigma Aldrich, St. Louis, MO, USA), respectively.

Skim milk samples were quantified at two different sample dilutions dedicated to the individual parameter, namely 25,000- and 50,000-fold diluted for S-IgA and 50,000- and 100,000-fold diluted for Lf, each in duplicate.

For the determination of SIgA, the primary mouse monoclonal anti-secretory component IgA antibodies (Sigma, St. Louis, MO, USA) were used. For detection, horseradish peroxidase (HRP) and alkaline phosphatase (AP)-labeled secondary antibodies were used, for SIgA goat anti-mouse IgG antibodies horseradish peroxidase-labeled (Sigma, St. Louis, MO, USA) and for Lf rabbit anti-human lactoferrin phosphatase-labeled antibodies (Jackson ImmunoResearch Europe Ltd., Ely, UK). The color reactions for HRP and AP were developed. Then, the absorbance was measured at 492 nm (reference filter 630 nm) for HRP and at 405 nm (reference filter 630 nm) for AP, respectively, using Synergy LX Multi-Mode Reader (BioTek Instruments, Inc., Winooski, VT, USA).

The details concerning the precision of the tests performed, the coefficients of variation for intra- and inter-assay, are as follows: For SIgA-ELISA 2.6% and 5.4%, respectively, and for Lf-ELISA 0.8% and 1.8%, respectively.

The human milk samples, before being analyzed for determination of Lf and SIgA, were not normalized to have the same total protein content.

### 2.6. CRP Concentration

The concentration of CRP in defatted HM samples was quantified by the enzyme-linked immunosorbent assay (ELISA). Anti-C-reactive protein antibody (IgG fraction of antiserum) (Sigma Aldrich, St. Louis, MO, USA) diluted 1:5000 in TBS pH = 8.0, and as a capture antibody was taken, the plates were incubated for 2 h at 37 °C. For the blocking step, TBS (pH 8.0) containing 0.1% Tween-20 and 0.2 mg% BSA was used, and the plates were incubated for 1 h at 37 °C and overnight at 4 °C. For testing, 100 μL of 20-fold diluted defatted milk samples and a standard preparation of C-reactive protein from human fluids from 0.08 to 5 ng/100 μL (Sigma Aldrich, St. Louis, MO, USA) in Tris-HCl with 0.05% Tween-20, pH = 8.0 were transferred to the wells of a microtiter plate (Nunc International, Naperville, IL, USA) and left for 1 h at 37 °C. Goat anti-CRP horseradish peroxidase (HRP)-labeled antibodies (Abcam, Cambridge Biomedical Campus, Cambridge, UK) diluted 1:50,000 in TBS with 0.05% Tween-20 were used as a detection antibody. The reaction was developed by adding orthophenylenediamine (Calbiochem, Denmark) in 0.1 M citrate buffer, pH 5.0 with H_2_O_2_, and the plates were incubated for 10 min at room temperature in the dark. The reaction was stopped with 12.5% H_2_SO_4_, and the absorbance was measured in a Synergy LX Multi-Mode Reader (BioTek Instruments, Inc., Winooski, VT, USA) at 492 nm, with 630 nm as the reference filter. All defatted milk samples were assayed in duplicate. The intra-assay and inter-assay coefficients of variation were 0.6% and 1.8%, respectively.

### 2.7. Anti-SARS-CoV-2 IgA Detection

The specific anti-SARS-CoV-2 IgA was measured by using cassette serological lateral flow detection tests. The kits were sensitive to the receptor-binding domain (RBD) of the SARS-CoV-2 spike protein and have been validated with clinical blood samples. Details about the tests and their specifications have been described by Zeng et al. [27,28].

**Figure 1:**
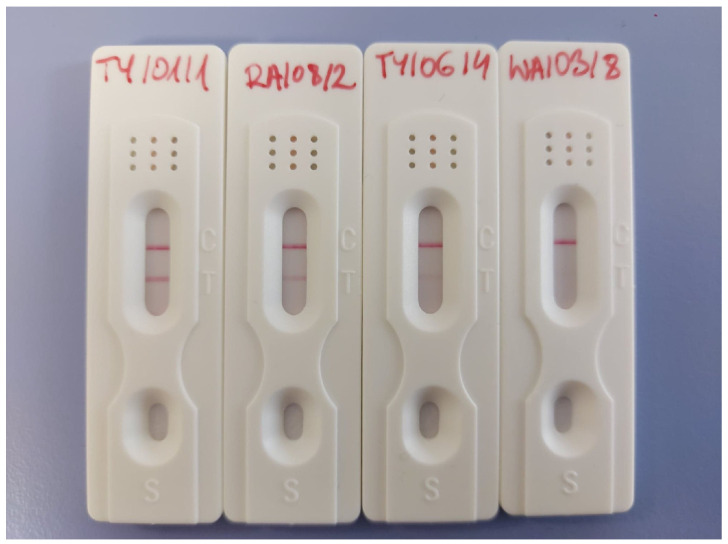
Scale of the results.

However, the quality assays did not allow for detecting IgA levels, but the sensitivity of tests enabled us to scale the results, as illustrated in Figure 1 and Table 1.

### 2.8. Statistical Analysis

The statistical analysis was performed with TIBCO STATISTICA ver. 13.3 (StatSoft, Inc., Tulsa, OK, USA). Qualitative variables such as the age of the mother (≤29, ≥30 years old), gestational age (preterm, term), and mode of delivery (vaginal birth, cesarean section) were presented as frequencies and percentages (% (n/*N*)). The Freeman–Halton test was used to compare groups (*p*-value lower than 0.05 was regarded as significant).

The Shapiro–Wilk test was used to estimate the normality of distribution (the *p*-value lower than 0.05 was considered as no normal distribution). Due to interindividual differences in the biochemical parameters of milk, in the analysis, nonparametric tests were used. The quantitative variables’ values were given as the median, mean ± SD (standard deviation) and additionally as the median with the twenty-fifth to seventy-fifth percentiles. Outlier analysis using the Tukey method showed the limits of typical observations, and to reduce model load, the outliers were reduced to limit values. The Mann–Whitney U test and the Kruskal–Wallis test were used for the calculation of statistical significance. A two-tailed *p*-value lower than 0.05 was regarded as significant.

## 3. Results

### 3.1. Characteristics of the Study and Control Group

Women from both groups were similar in demographic and perinatal characteristics (Table 2). The average age for the control group was 30.90 ± 3.75 years, while for the study group, it was 30.95 ± 5.52 years. Most of the women gave birth to full-term babies. Only two women from the study group and one from the control group gave birth prematurely (≤37 weeks). No significant differences were found for maternal and gestational age between study subgroups (*p >* 0.05). Three-quarters of the enrolled in the COVID-19 group of women delivered by cesarean section, while this type of delivery mode constituted the uninfected group 100% (*p* = 0.047). In total, 3 out of 20 mothers from the study group suffered from hypothyroidism. In this group, 21 newborns were born, including one pair of twins. All children were born healthy, which was confirmed by PCR tests for COVID-19 performed in the first days of their lives.

**Figure 2:**
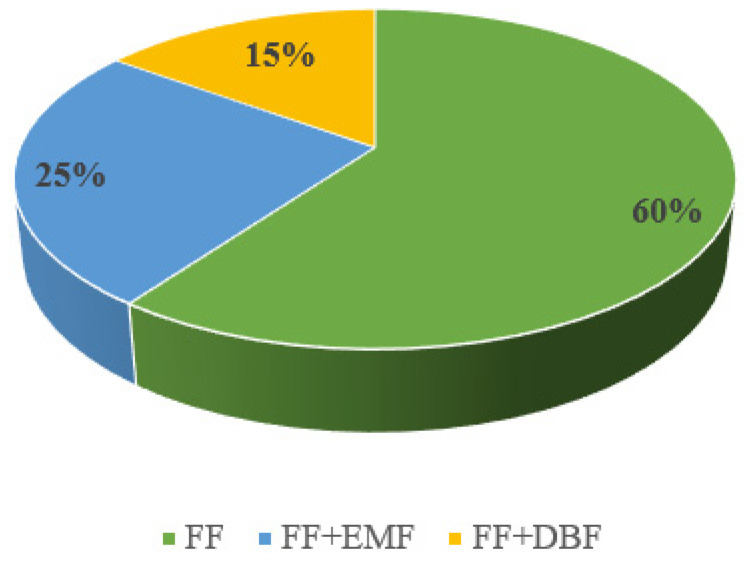
Model of feeding by mothers from the study group. FF—Formula feeding, EMF—Expressed milk feeding, DBF—Direct breastfeeding.

Due to the recommendations in Poland during the study period, breastfeeding was not advised for mothers infected with COVID-19. As a result, after recovering from the illness, the mothers from the study group were asked about their infant feeding methods and lactation stimulation. Most women (90%, *n* = 18) admitted that they started stimulation of lactation during their hospitalization. All of them began to pump on the first or second day postpartum. Figure 2 contains detailed information about feeding newborns after being transferred to a rooming-in or discharged home. All infants were fed with formula (fully or partially) and 60% (*n* = 12) did not receive any amount of mother’s own milk.

### 3.2. Proteins Concentrations in Skim Milk Samples

**Figure 3:**
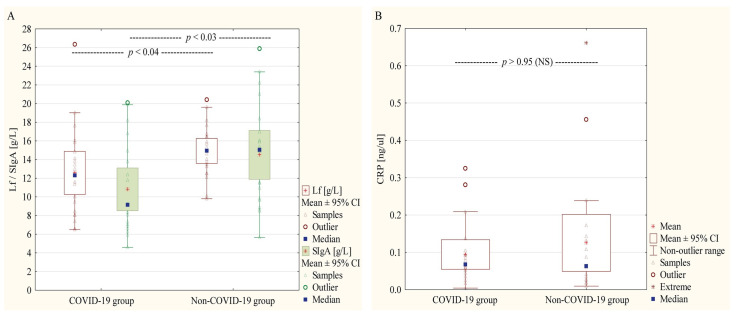
Comparison of lactoferrin (Lf) and secretory IgA (SIgA) (**A**), and C-reactive protein (CRP) (**B**) concentrations in milk samples collected from mothers with COVID-19 and uninfected mothers (Control group). Data are given as median and mean ± 95% Confidence Interval (CI). NS—not significant.

There were no statistically significant differences in the mean concentration of CRP in the tested samples (*p <* 0.95). For the remaining parameters, statistically significant differences were observed. In the study group, there were lower concentrations of Lf (*p <* 0.04) and SIgA (*p <* 0.03) (Figure 3).

**Figure 4:**
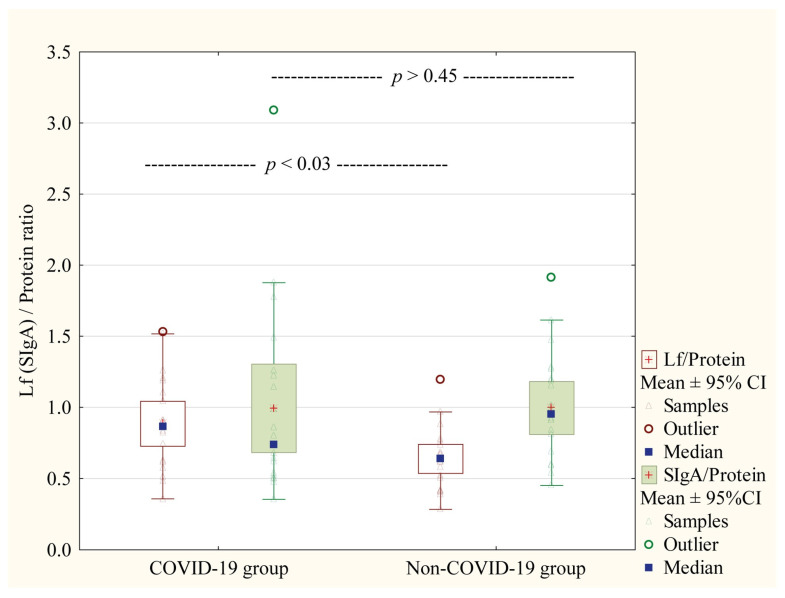
Comparison of Lf/Protein and SIgA/Protein ratio between COVID-19 cohort and uninfected mothers (Control group). Data are given as median and mean ± 95% Confidence Interval (CI). NS—not significant.

Figure 4 presents the Lf/Protein and SIgA/Protein ratio between the COVID-19 cohort and healthy women. We did not observe statistically significant differences in the case of SIgA/Protein (*p >* 0.45). The statistically significant differences were observed between the study and control groups for the Lf/Protein ratio, which was higher in the milk of women with COVID-19 at delivery (*p <* 0.03).

Most of the women from the study group (75%, *n* = 15) had a positive test result for the presence of antibodies directed against virus proteins in the colostrum (IgA anti-RBD), in 10% (*n* = 2) the results were negative, in 15% (*n* = 3) the results were unknown. Most of the uninfected mothers (90%, *n* = 18) had a negative result on the cassette test, and for the rest (10%, *n* = 2) the results were inconclusive/unknown.

**Figure 5:**
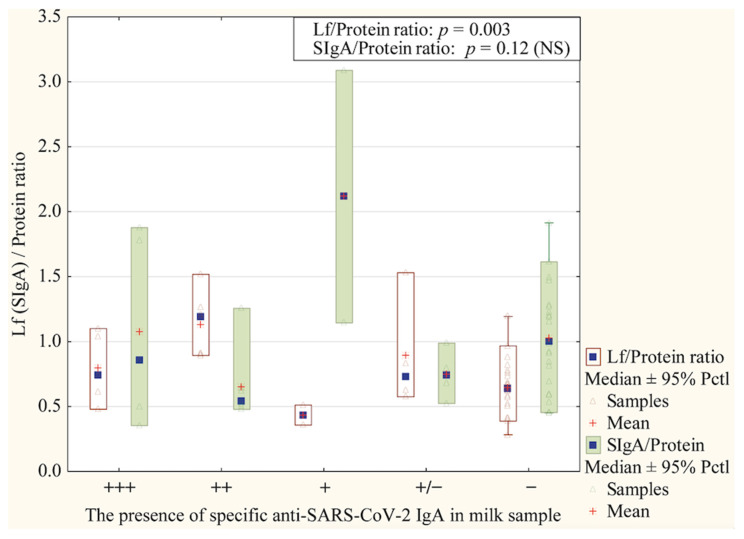
Comparison of Lf/Protein and SIgA/Protein ratio in milk samples collected from mothers with COVID-19 about the result of cassette serological lateral flow detection tests on the presence of the specific anti-SARS-CoV-2 IgA. Data are given as mean and median ± 95% Percentile (Pctl). NS—not significant.

Figure 5 shows a comparison of the dependence of the levels of immune factors and total protein ratio on the presence of specific anti-SARS-CoV-2 IgA. There was a statistically significant impact of the Lf/Protein ratio (*p =* 0.003). A higher Lf/Protein ratio was observed in the milk of women who received very noticeable or low-level results compared to those with negative or unknown results. In the case of the SIgA/Protein ratio (*p =* 0.12), there was no statistically significant difference.

**Figure 6:**
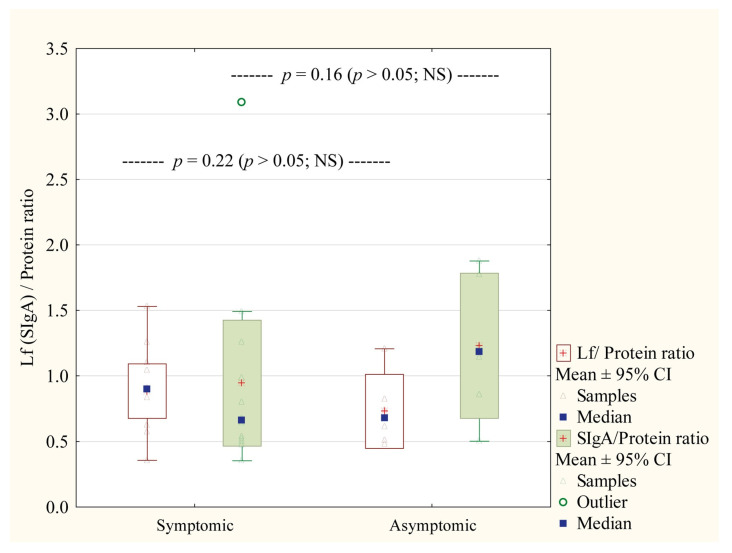
Comparison of Lf/Protein and SIgA/Protein ratio in milk samples collected from symptomatic and asymptomatic mothers with COVID-19. Data are given as median and mean ± 95% Confidence Interval (CI). NS—not significant.

Furthermore, Lf/Protein and SIgA/Protein ratios were compared between symptomatic and asymptomatic mothers in the study group (Figure 6). There were no statistically significant differences in either case.

## 4. Discussion

Breastfeeding practices were severely impacted by the COVID-19 pandemic, especially during the first months of the SARS-CoV-2 virus spreading worldwide [2,29]. In Poland, the first guidelines for perinatal care on the obstetrical population were published at the beginning of the pandemic in March 2020 and were very restrictive [30]. Primarily, the knowledge level regarding the transmission of the virus from the mother to the newborn was fragmentary; therefore, the separation of the mother–child dyad was recommended at the time of receiving a negative PCR test [30]. In the following months of the COVID-19 pandemic, the knowledge level regarding perinatal care for mothers and offspring was expanded upon. Giuliani et al. [31] analyzed the association between breastfeeding and offspring’s positive results of COVID-19 tests. They showed that the natural way of feeding was not related to an increased risk of virus transmission from women to newborns in settings where mothers used preventive measures, e.g., wearing masks and washing their hands. The systematic review by Graciliano et al. [2]. demonstrated that maternal SARS-CoV-2 exposure enhances breast milk’s immunological components, including neutralizing antibodies like IgA and IgG, which inhibit viral infectivity while maintaining safety for infants.

In this study, we report that SIgA and Lf were present in the HM samples of mothers suffering from COVID-19. Moreover, the presence of anti-SARS-CoV-2 IgA in their milk was confirmed, indicating that breastfeeding is an essential part of supporting the newborn’s immunity during an early stage of postnatal life. This study shows that maternal stress related to COVID-19 can affect the level of SIgA and Lf in HM. The data do not show a direct relationship between Lf, SIgA, and Lf/Protein and SIgA/Protein ratios regarding maternal severity of COVID-19 disease. Moreover, we did not observe significant differences in CRP levels in milk samples from mothers with active status of SARS-CoV-2 infection. This study showed the human milk susceptibility to alteration due to the mother’s health status, namely the presence of viral infection. Colostrum contains the highest concentrations of SIgA and Lf, providing a concentrated defense during the first days of life, and their concentrations have been reported to range from 1.5 to 83.7 g/L [32,33] and 5–52.82 g/L [34,35,36], respectively. In our study, we demonstrated that SIgA concentration in colostrum samples collected from mothers with COVID-19 was 9.15 (range: 6.95–14.36) g/L in the study group and was significantly lower than in the control group (15.01 g/L, range: 9.71–17.67 g/L) (Figure 3). Similarly, Lf concentration in the milk of COVID-19-positive mothers obtained 12.30 (8.24–15.33) g/L and was significantly lower than for uninfected mothers (14.95 (12.90–16.60) g/L) (Figure 3). The concentration of SIgA and Lf in HM varies between mothers and changes throughout lactation [24,26,33,35]. This variation is influenced by numerous maternal, environmental, and geographical factors [12,15,26,34,37]. It is worth pointing out that maternal psychological and social factors also affect SIgA and Lf concentration in HM. It was found that maternal anxiety was negatively associated with milk levels of SIgA (β = −0.30, *p* = 0.004) and Lf (β = −0.23, *p* = 0.028) [18]. The maternal emotional states exert distinct impacts on the immune properties of HM [38,39,40]. Perceived stress correlates with reduced immune defense, marked by lower concentrations of secretory immunoglobulin A (SIgA), a critical antibody for infant immunity. Conversely, elevated anger scores, measured via the Profile of Mood States (POMS) questionnaire, were associated with higher SIgA levels, indicating a potential immune-boosting effect [38,39]. The complex interplay between maternal psychological factors and HM composition was summarized in a review by Skowron and coworkers [40], and it underscores the need for holistic maternal care to optimize infant health outcomes. As observed in this study, the lower concentration of total SIgA in the HM samples from mothers infected with SARS-CoV-2 in comparison to uninfected mothers with negative tests for specific anti-SARS-CoV-2 antibodies may be the result of stress related to COVID-19. During the worldwide pandemic, a part of the obstetrical population experienced the distressing reality of delivering alone and the disturbance of the mother–infant dyad relationship [41]. In a Greek study conducted in 2019, women who experienced postpartum stress had increased cortisol levels in saliva and lower IgA levels in milk [22]. Moreover, Moirsgenti et al. [22] concluded that the mother’s stress, which negatively affects the immunological factors of her milk, may harm the offspring’s well-being. 

Another factor that may influence the results presented in this study is the mode of delivery. Previous research has shown that the type of delivery can affect the nutrient composition of human milk, particularly its protein and fatty acid content [42,43]. The authors speculated that the hormonal activity associated with vaginal delivery might lead to changes in the protein composition of human milk, thereby supporting the optimal development of key physiological functions in newborns. Other factors known to impact lactoferrin (Lf) concentration in human milk include maternal race, age, body mass index (BMI), parity, and preterm delivery [35,44]. Interestingly, while the mode of delivery (cesarean vs. vaginal) has been shown to influence the concentration of the free secretory component (SC) of IgA in human milk, it does not appear to affect SIgA itself [45].

The study found that lactoferrin (Lf) concentrations in colostrum were lower in SARS-CoV-2 positive mothers (median: 12.30 g/L, range: 8.24–15.33 g/L) compared to uninfected mothers (median: 14.95 g/L, range: 12.90–16.60 g/L) (Figure 3). This difference aligns with previous research by Trofin et al. [46], which reported similar reductions in Lf levels associated with COVID-19 infection during lactation. In contrast, Briana et al. [47] observed similar levels of Lf in colostrum samples on day 3 postpartum; however, in the milk of symptomatic mothers, the level of Lf was not significantly lower than in asymptomatic women. On the other hand, Gaweł et al. [36] showed that the colostrum Lf concentration during active SARS-CoV-2 infection was higher (58 g/L, range: 41–75 g/L) than in colostrum samples from post-infected COVID-19 mothers (40 g/L, range: 31–56 g/L). Observed differences in Lf concentration in colostrum samples might be associated with the size of the analyzed groups. It should be emphasized that the study groups in Brianna et al. [47] and Gaweł et al. [36] obtained 13 and 12 mothers during active infection COVID-19, respectively, and they were smaller than in this study (*n* = 20), which probably signed into a higher risk of random variability, lower precision and reliability levels. It was assumed that during severe/acute infections with strong clinical manifestation, the immune system of women adapts to protect mother–infant dyad, potentially increasing the level of immune factors like Lf in HM to enrich the infant’s passive immunity [48]. The authors [36,49] reported that a lower level of Lf in milk collected from symptomatic mothers was associated with a “compensatory decrease” of this antiviral biomolecule, which can interact with viruses and/or bind heparan sulfate proteoglycans on the host cell surface. This hypothesis was confirmed in our and Trofin et al. [46] data. The authors [46] analyzed the milk samples from COVID-19-infected (*n* = 24) and vaccinated mothers (n = 26) and suggested that a decrease in Lf is the net result of immunization effects (in the way of “natural” exposure to infection or getting vaccination), further supporting the dynamic nature of immune responses in the maternal–infant dyad. Currently, discussion of Lf to strengthen resistance against respiratory tract infections is underway [49,50,51,52,53,54]. A meta-analysis performed by Ali et al. [17] showed that nutrients inspired by HM, especially Lf supplementation, can enhance the immune response to viruses and/or prevent direct interaction of viral molecules with host cells, which might be evidence that favors Lf fortification of infant formula. 

In this study, we detected in colostrum samples the presence of the specific anti-SARS-CoV-2 IgA, which is in line with data presented previously [55,56,57]. The detection aligns with findings of mucosal immunity transfer via breastfeeding, but variability in IgA levels across studies suggests complex interactions between maternal immune responses, vaccination status, and timing of sample collection relative to infection [2,13,36,55,56,57]. A current knowledge state regarding the impact of maternal SARS-CoV-2 infection and/or anti-COVID-19 vaccination on human milk immunological components was summarized by Graciliano et al. [2].

The authors [58,59] reported that HM samples collected from COVID-19-positive mothers have higher levels of SARS-CoV-2-specific IgA and IgM antibodies capable of eliciting neutrophil phagocytic clearance, neutralization effect, and promoting mucosal immunity. A later study by Hochmayr and coworkers [60], in which milk from 140 women was tested, demonstrated that HM from women with severe clinical manifestations of COVID-19 disease has higher levels of IgA and IgG anti-S1RBD of SARS-CoV-2 in comparison to asymptomatic or mild symptomatic women. In this study, in contrast to data presented by Hochmayr et al. [60], we did not observe significant differences in the concentration of SIgA (*p* > 0.38) and Lf (*p* > 0.55). Moreover, the comparison of ratios for the analyzed immunological components levels to total protein (Lf/Protein and SIgA/Protein ratios), showed no significant difference between symptomatic and asymptomatic subgroups (Figure 6). Interestingly, a significant difference between analyzed groups was noted for Lf/Protein ratio (*p* = 0.03), but not for SIgA/Protein, due to the presence of the specific anti-SARS-CoV-2 IgA in colostrum (Figure 5). The higher Lf/Protein ratio was in the group of COVID-19-infected mothers, and three-quarters of women from this group had positive results on the test for the presence of the specific SIgA in the colostrum. Our data are aligned with Briana et al.’s [47] suggestion that colostral Lf level can be affected by the severity of maternal COVID-19 infection. 

Additionally, in the presented study, we analyzed the level of CRP in HM samples; however, no significant differences were noted concerning the presence of COVID-19 in mothers. To the best of our knowledge, our findings are the first that characterize the concentration of CRP in COVID-19-positive mothers’ milk during early lactation, with additional emphasis on the severity of the clinical manifestation of the disease. Chen et al. [61] reported that plasma CRP level is positively associated with the severity of COVID-19. A later study [62] demonstrated that serum CRP levels might be a predictor of the severity and development of sickness in patients with COVID-19 infections. In contrast to the maternal plasma, in HM samples we did not observe statistically significant differences in the CRP concentrations between groups of women with positive and negative results against SARS-CoV-2. Moreover, we did not report statistical significance in CRP levels with the severity of symptoms in a group of COVID-19-positive mothers. No significant changes in the level of human milk CRP in women are aligned with data presented by Fetherston et al. [63], who did not observe a significant increase in CRP level in HM throughout the mastitis episode. Landau-Crangle et al. [64] found that CRP is positively associated with inflammatory states, such as overweight and obesity; however, in our study, this anthropometric factor was not analyzed. Despite that the consequences of offspring receiving altered concentrations of HM inflammatory markers are unknown; however, we contribute to the Whitaker et al. [65] hypothesis that there are implications for the intergenerational transmission of disease risk.

### Strengths and Limitations

The undeniable strength of the present study is the evaluation of innate and adaptive bioactive component levels in HM samples about the ongoing SARS-CoV-2 infection in women and the severity of COVID-19 disease. Further strengths of the study include a higher sample size than other studies presented so far, which might translate into a lower risk of random variability, and higher precision and reliability levels. The standardized protocol used for sample collection during the COVID-19 pandemic allowed us to create an as-much-as-possible homogeneous cohort of lactating women concerning women outcomes, which limits potential confounding factors. Additionally, it should be emphasized that both groups were collected during the same period, namely during the ongoing COVID-19 pandemic, which was a difficult experience for the obstetrical population.

Finally, several limitations of our study should be pointed out. Firstly, the sample size, despite being higher than other studies, was lower than the minimal sample size (75 participants), which could allow an obtain confidence interval of 5% and a level of confidence of 95%. In this study, the sample size bias was calculated at the level of 9.3%. The second limitation might be the collection of milk samples included in the control group only in one hospital, while the milk samples collected from COVID-19-positive mothers enrolled in the study group were collected in different geographical localization of Poland in hospitals designated for perinatal care of mothers with COVID-19 in the postpartum. The results obtained for analyzed groups introduce several limitations, including limited generalizability, exploration of heterogeneity and selection bias. Another limitation of this study is the absence of data on maternal psychological status. Milk samples from both COVID-19-positive and uninfected mothers were collected during the most challenging phase of the COVID-19 pandemic, a time when breastfeeding was strongly discouraged. The study design focused on comparing Lf and SIgA concentrations between groups, taking into account general maternal characteristics and obstetrical variables. However, we did not anticipate that maternal fear and anxiety might significantly influence the biochemical parameters analyzed, and as a result, psychological data were not collected. Additionally, the uninfected control group consisted exclusively of mothers who delivered by cesarean section, which may introduce selection bias and limit the generalizability of the findings. The performed statistical analysis revealed the alterations in the composition of colostrum samples during ongoing maternal COVID-19 that are assumed to be sufficient to verify the study thesis, and although the obtained results provide an overview of the addressed issue, there is still a need to confirm these results with a larger group of lactating mothers. Future studies are needed to evaluate the potential impact of perinatal stress, and psychological and socioeconomic status on bioactive components of HM.

## 5. Conclusions

The data presented in this study show the dynamism of maternal milk’s composition and its susceptibility to alteration due to the mother’s health status, including viral infections. Moreover, being the first, we report that maternal plasma parameters of infection do not translate into higher levels of CRP in HM. Considering the impact of maternal infections on the mother–infant dyad, maternal health should be considered in a multi-faceted approach as a factor affecting the well-being of the next generations. Understanding these factors is vital for optimizing infant nutrition and immunity, particularly for vulnerable populations, e.g., preterm infants. It is an argument for supporting women in breastfeeding even in the case of active infection. The COVID-19 pandemic should be a lesson for professionals and an opportunity to develop recommendations to determine how to behave in such a crisis properly. As was reported by authors [58], the pattern of immunoglobulins during maternal infection and/or vaccination can provide essential insights for the design of next-generation vaccines or pharmaceuticals to protect the mother–infant dyad. However, further well-designed, longitudinal research in this area is needed. Our study might support future investigations focusing on providing evidence that Lf may have a beneficial impact on the protection and/or treatment of some viral diseases, and might favor Lf fortification of infant formula.

## Figures and Tables

**Table 1:** Scale of the results.

(−)	No detection
(+/−)	Unknown
(+)	Low level
(++)/(+++)	Very noticeable

**Table 2:** Characteristics of the study population.

	COVID-19-Positive Mothers *N* = 20 (% (*n/N*))	COVID-19-Negative Mothers *N* = 20 (% (*n/N*))	*p*-Value
Race/ethnicity Caucasian	100% (20/20)	100% (20/20)	NA
Maternal age (mean ± SD)	30.95 ± 5.52 (median: 31)	30.90 ± 3.75 (median: 31)	0.75 (NS)
≤29	35.0% (7/20)	45.0% (9/20)	
≥30	60.0% (12/20)	55.0% (11/20)	
no information	5.0% (1/20)	0.0% (0/20)	
Gestational age (mean ± SD)	38.56 ± 1.46 (median: 39)	40.35 ± 1.27 (median: 41)	0.41 (NS)
preterm (≤37 weeks)	10.0% (2/20)	5.0% (1/20)	
term (38–41 weeks)	80.0% (16/20)	95.0% (19/20)	
no information	10.0% (2/20)	0.0% (0/20)	
Delivery mode			0.047
vaginal birth	15.0% (3/20)	0.0% (0/20)	
cesarean section	75.0% (15/20)	100.0% (20/20)	
no information	10.0% (2/20)	0.0% (0/20)	

NS—not significant; NA—not analyzed.

## Data Availability

The raw data supporting the conclusions of this article will be made available by the authors upon request.
